# Genetically modified crops in Africa: progress, challenges, and future prospects

**DOI:** 10.1080/21645698.2026.2746154

**Published:** 2026-10-21

**Authors:** Samuel Oluwasegun Ipinyomi, Aminu Aliyu, Chiamaka Chinasa Obasi, Lilian Noela Wesonga, Wagatua Njoroge, Joseph Ayitey Nunoo, Matilda Owusu-Ansah, Rukiya Danful, Wilfred Abincha, Nana Mohammed Opuni, Richard Katembo Kataliko

**Affiliations:** a Horticultural Sciences Department, University of Florida, Gainesville, FL, USA; b Department of Botany, Faculty of Life Sciences, Ahmadu Bello University, Zaria, Nigeria; c Department of Crop Science and Horticulture, Nnamdi Azikiwe University Awka, Awka, Nigeria; d Department of Water and Agricultural Resource Management, University of Embu, Embu, Kenya; e Kenya Agricultural and Livestock Research Organization (KALRO), Industrial Crops Research Centre, Mwea, Kenya; f Biotechnology and Nuclear Agriculture Research Institute (BNARI)-Ghana Atomic Energy Commission (GAEC), Accra, Ghana; g Premium Foods Limited, Ejisu-Kwaso, Ghana; h West African Center for Crop Improvement, University of Ghana, Accra, Ghana; i Research and Development Department, Kentegra Biotechnology Holdings LLC, Nairobi, Kenya; j Faculty of Agricultural Sciences, Université Catholique du Graben, Butembo, Democratic Republic of Congo

**Keywords:** Food security in Africa, genome editing, public perception, sustainable agriculture, transgenic crops

## Abstract

Genetically modified (GM) crops represent a promising solution to address the challenges of food security and economic growth in Africa. This review provides a comprehensive analysis of the current state of GM crop adoption in Africa, highlighting the achievements, obstacles, and prospects. Case studies from nine African countries, namely South Africa, Burkina Faso, Sudan, Egypt, Kenya, Ethiopia, Nigeria, Uganda, and Ghana, illustrate the diverse approaches and outcomes of GM crop cultivation in different African contexts. Key factors influencing adoption include biosafety regulation, institutional capacity, public perception, seed systems, market considerations, stakeholder engagement, and regional policy coordination. The review also highlights the potential of emerging technologies, including genome editing and gene stacking, for developing climate-resilient and nutritionally improved crops. To fully realize the potential of GM technology in Africa, stakeholders must collaborate to establish consistent, science-based regulatory frameworks, raise public awareness, foster public-private partnerships, support farmer-oriented research, and strengthen regional coordination.

## Introduction

Global population growth and declining arable land demand require increased food production, prompting the use of modern biotechnological techniques to support global food security. The production of genetically modified (GM) crops has increased rapidly in both developing and developed countries. The adoption of GM technology has led to a 22% increase in crop yield and a 37% reduction in insecticide use, resulting in a 68% increase in profits for farmers due to reduced production costs.^1,2^

Genetically modified crops play a critical role in global food security because of their economic importance. Zhang et al.^3^ reported a global increment in farm income of $116 billion, which was attributed to GM food within a period of six years (2006–2012). Forty-two percent of this growth was due to increased yield, while 58% was due to reduced production cost because of the use of GM varieties that are resistant to weeds and pests; therefore, herbicides and pesticides were not used.^3^

Plants can be genetically transformed through two principal approaches: direct and indirect genetic transformation. Direct transformation involves the physical delivery of foreign DNA into plant cells or tissues. One example is particle bombardment, in which DNA-coated tungsten or gold microparticles are accelerated into target tissues at high velocity, facilitating the introduction of DNA into plant cells.^4,5^ Indirect genetic transformation is principally mediated by *Agrobacterium tumefaciens*, which naturally transfers a segment of DNA known as transfer DNA (T-DNA) into plant cells. In engineered transformation systems, the T-DNA region is delimited by right and left border sequences, and the DNA between these borders can be modified to carry the gene of interest. The tumor-inducing gene systems remove or inactivate the tumor-inducing genes, while the Agrobacterium-mediated transfer machinery delivers the recombinant T-DNA into the plant cell. The introduced T-DNA may subsequently integrate into the plant genome, resulting in genetic transformation.^6,7^ CRISPR-Cas9, in contrast, is a genome-editing technology that can be delivered into plant cells through direct or Agrobacterium-mediated transformation systems to introduce targeted genetic modifications.^3^

Genetically modified crops are improved for various traits, such as shelf life, resistance to abiotic and biotic stresses, phytoremediation, and improved nutritional values.^2^ In addition to these agronomic and nutritional benefits, the adoption of GM crops worldwide has contributed to a reduction in the use of certain pest-control and herbicide-related chemicals, with associated environmental benefits such as reduced pesticide runoff, lower chemical exposure, and improved soil-health outcomes in some production systems.^8^ Despite the economic importance and potential health, nutritional, and environmental benefits of GM crops, their development and consumption have generated continuing public debate and concerns regarding food safety, environmental risks, ethics, and socioeconomic implications.^3^ Public perceptions of GM crops are influenced by several interacting factors, including limited understanding of genetic modification, inadequate or ineffective public communication, uncertainty about long-term risks, mistrust of regulatory institutions and biotechnology companies, and the influence of media coverage and advocacy campaigns.^1,3^ In addition, concerns regarding corporate control of agricultural biotechnology, intellectual property rights, farmer autonomy, and the possible concentration of control over seeds and food production have contributed to public apprehension in some regions, particularly in Europe. These concerns should not be dismissed as arising solely from a lack of scientific knowledge; rather, they reflect broader questions about transparency, accountability, public participation, risk governance, and the distribution of the benefits and risks associated with GM technology.^9–12^

In Africa, the impact of GM crops is increasingly evident. Notable success stories include the commercial cultivation of Bt cotton in Burkina Faso, which initially increased yields and reduced pesticide use,^13^ and the adoption of insect-resistant maize in South Africa, which has improved both productivity and farmer livelihoods.^14^ However, these examples also highlight the importance of robust regulatory oversight and adaptation to local contexts, as seen in the eventual withdrawal of Bt cotton in Burkina Faso due to fiber quality concerns.^15^

The main challenges facing GM crops in Africa include ensuring efficient, environmentally safe, and effective production, as well as the commercial release of these crops while improving food security (Figure 1). For GM crops to be fully accepted in Africa, there is a need for good collaboration between biosafety regulatory authorities and variety release authorities. Although newly developed GM crops may be directly released for commercial production by farmers, most of the time they are introgressed into locally adapted, farmer-preferred non-GM varieties already being cultivated.^16^ Looking ahead, advances in genome editing, the development of region-specific GM traits, and increased public engagement offer promising prospects for expanding the benefits of GM technology across the continent.

**Figure 1.**
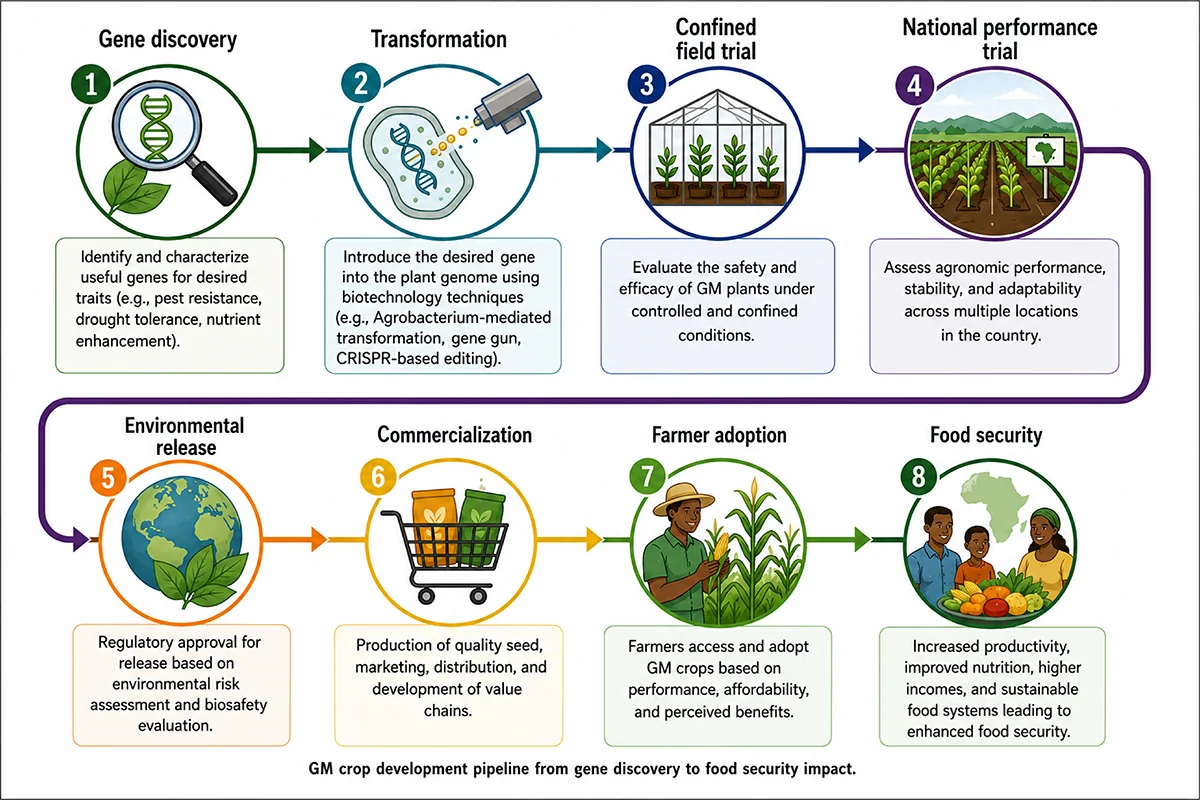
GM crop development pipeline from gene discovery to food security impact.

Therefore, this review aims to provide a comprehensive analysis of the current state of GM crop adoption in Africa, highlighting both the achievements and obstacles encountered. It explores the regulatory frameworks, public perception, environmental impacts, and agricultural policies that influence the implementation of GM technology across the continent. Additionally, the review will examine case studies from various African countries to illustrate the diverse experiences and prospects of GM crops on the continent.

## African Countries with Notable GM Activity

### South Africa

South Africa became the first African country to commercialize genetically modified (GM) crops in 1997 following approval of Bt cotton.^17^ Since then, the country has developed one of the continent’s most mature biosafety systems under the Genetically Modified Organisms Act, allowing science-based evaluation and commercialization of GM crops. South Africa remains the only African country with widespread commercial production of GM staple crops, including maize and soybean.^18,19^ South Africa is therefore perceived as a model African country that has effectively allowed the use of GMOs for commercial purposes (Figure 2).

With the first release of Bt cotton, other GM crops have been commercialized with passage of time. MON 810 was the first Bt maize first released in South Africa and was commercialized by the brand name “Yieldgard.” However, it failed insect resistance and was soon replaced by MON 89,034 in 2010.^20,21^ The drought-tolerant (DT) maize, MON 87,460, following several multi-year field evaluations from 2007, was finally approved and released under the brand name “DroughtGard.” This variety was tested in 2017 by a limited number of smallholders in South Africa under the Water Efficient Maize for Africa (WEMA) project.^22^

South Africa has consistently recorded the highest GM crop adoption rates in Africa. Commercialization has resulted in higher maize and cotton yields, lower insecticide use, increased farm profitability, and improved food security. More than two decades of GM crop consumption have also provided no documented evidence of adverse human health effects.

Despite its success, insect resistance evolution (e.g., MON810) emphasizes the importance of integrated pest management and resistance stewardship. Future priorities include gene stacking, climate-resilient crops, and genome-edited varieties.

### Burkina Faso

In 2015, Burkina Faso became Africa’s leading Bt cotton producer, overtaking South Africa. This dominance largely stems from the prevalence of small-scale farmers in Burkina Faso’s cotton sector. The country’s cotton industry is marked by a well-regulated, centralized system that facilitates farmers’ access to reliable credit facilities. Cotton companies supply essential farming inputs, such as seeds and fertilizers, to the farmers, who then sell their cotton back to these companies.^17^

Burkina Faso cotton is renowned for its high quality, with premium long, strong, uniform fibers and a high ginning ratio that strengthens its reputation in the cotton industry. However, cotton quality declined after Monsanto successfully introduced Bt traits into locally adapted cultivars. The new introgressed cultivars, although requiring six times less pesticide than conventional cotton, exhibited undesirable characteristics, including reduced staple length and ginning ratio, that persisted for an extended period without a clear cause.^23^ During the 2013/2014 cropping season, Bt cotton produced shorter fiber lengths than traditional varieties, marking a 40% decline in quality since the 2005/2006 season. This deterioration in quality adversely affected the market value and reputation of Burkina Faso’s cotton. Breeders struggled to explain this decline, as introgressed offspring are generally expected to retain all traits except the introgressed one. Consequently, Burkina Faso’s cotton companies, operating within a centralized system, decided to discontinue Bt cotton and planned to remove it completely by the 2017/2018 cropping season.^17^ The situation in Burkina Faso raised significant questions regarding the commercial production of GMOs in Africa. Firstly, the Bt cotton-breeding program had a limited scope and attempts to broaden it by combining insect resistance with other cotton qualities led to a decline in quality. Secondly, GMOs can generate conflicting interests among stakeholders. For instance, private companies and non-farmers decided to phase out Bt cotton, despite farmers’ satisfaction with yield and increased income. Conversely, private companies were dissatisfied with the poor cotton quality, which resulted in lower market prices. The phasing out of Bt cotton in Burkina Faso has influenced other African countries’ deliberations on adopting GMOs.

### Sudan

Sudan’s first biotechnological crop is insect-resistant (IR) Bt cotton, particularly the Seeni 1 variety, which was commercialized for both irrigated and rainfed regions.^24^ Seeni 1 is a Chinese Bt cotton genotype (*Gossypium hirsutum*) that incorporates the *Cry1A* gene from *Bacillus thuringiensis* (Bt). The National Variety Release Committee approved this variety in March 2012, followed by authorization for commercial production by the Biosafety Authority in June of the same year.^25^ In 2016, the range of commercially available Bt cotton materials expanded with the release of two Indian hybrids, KCH 1947 (Hindi 1) and JKCH 1050 (Hindi 2), both carrying the *Cry1Ac* gene.

From a modest 20,000 hectares in 2012, Bt cotton cultivation increased to 240,000 hectares in 2018, representing about 95% of total cotton-cultivated land.^22,26,27^ By the 2020/2021 production season, approximately 98% of the area cultivated for cotton was reportedly planted with Bt varieties, with similarly high adoption among cotton farmers (Figure 2).^28,29^ Sudan’s cotton exports averaged approximately USD 156.3 million during the period considered by Kedisso et al.^29^ Nevertheless, the magnitude of the economic benefits attributable specifically to genetic modification should be interpreted cautiously, as cotton production and export performance are also influenced by market prices, agronomic management, irrigation, access to inputs, and broader economic conditions. The country’s biotechnology pipeline is also moving beyond single- gene insect resistance. In response to evolving pest and weed-management challenges, trials are ongoing to test hybrid cotton carrying triple-gene traits for *Cry1Ac* and *Cry2A* and the glyphosate-herbicide-tolerant trait.^29^

**Figure 2.**
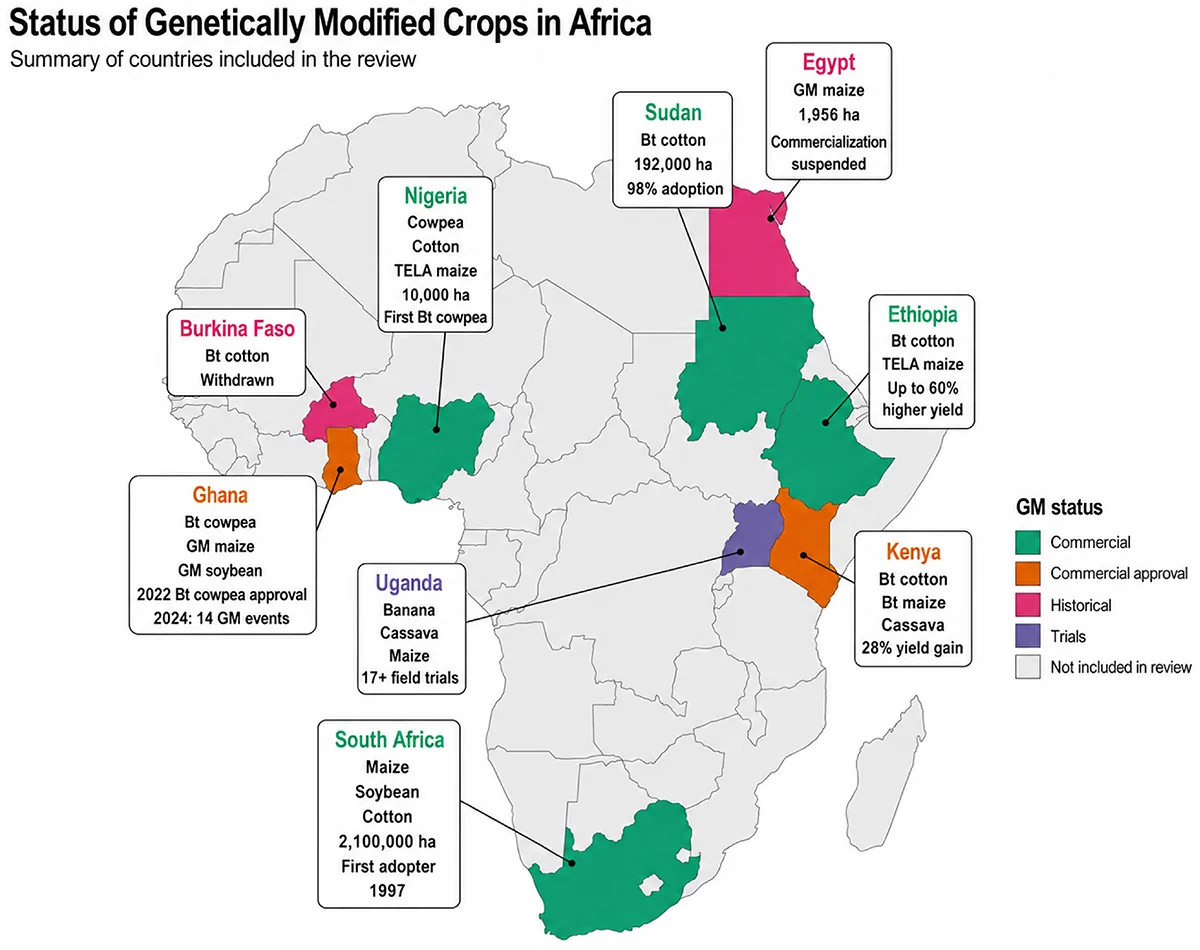
Status of genetically modified crops in Africa.

Despite high Bt cotton adoption, important institutional constraints remain. Kedisso et al.^29^ identified limitations in domestic research capacity, biosafety implementation, and institutional infrastructure, which may partly explain why commercial GM cultivation in Sudan has remained largely concentrated in cotton. Furthermore, the sector has depended substantially on imported Chinese and Indian genetic materials and private- sector participation, including contracting farmers to expand cultivated land area. Public–private partnerships may provide an important mechanism for combining private-sector investment and technology access with public-sector breeding, regulation, stewardship, and farmer-oriented research.^29^

### Egypt

Egypt was among the early African countries to commercialize genetically modified (GM) crops. Bt maize (MON810) was approved for commercial cultivation in Egypt in 2008 following field evaluation and biosafety assessment,^30–32^ with approximately 700 hectares initially planted.^18,32^ However, commercial cultivation was suspended in 2012 amid a combination of regulatory challenges, political uncertainty, and public concerns surrounding GM technology.^19,31,33^ At the time, Egypt lacked a comprehensive biosafety law, and the existing regulatory system had limitations in coordination and implementation.^31,34^ Concerns about public trust and the growing influence of multinational biotechnology companies also shaped Egypt’s approach to GM crop commercialization.^31^ In particular, Monsanto, the developer of the Bt maize technology, was perceived as having substantial influence over the technology and its commercialization, contributing to concerns about corporate control and trust in the public–private partnership.^31^

Economic and institutional considerations also influenced Egypt’s cautious approach.^35,36^ Worries about the potential effects of GM crop cultivation on agricultural exports to European markets, important destinations for Egyptian horticultural products, encouraged a more restrictive position.^36–38^ Institutional controversies surrounding the authorization and oversight of GM maize imports, further contributed to uncertainty about the early commercialization process. For example, a shipment of Bt maize was seized in 2012 because it had not received the required environmental approval.^39^ Public and media opposition also shaped perceptions of GM technology, particularly regarding potential health and environmental effects.^35^

Despite suspending commercial cultivation of GM crops, Egypt continued to permit imports of approved GM commodities, particularly maize and soybean for feed.^40^ The country has also maintained its biotechnology research capacity, including research involving genetic engineering and genome editing.^40,41^ Egypt is also a Party to the Cartagena Protocol on Biosafety, which it ratified in 2003.^42^ Egypt’s experience shows that successfully deploying GM crops requires more than scientific and biosafety assessments. Effective regulation, institutional trust, consideration of market interests, responsible corporate involvement, and meaningful stakeholder engagement are also essential.^43^

### Kenya

In 2009, Kenya enacted the Biosafety Act to allow the commercial cultivation of GM crops in principle; however, such crops were not placed on the Kenyan market.^44,45^ Later, in 2012, the Kenyan government banned the importation of GMO products; however, despite the ban, the government still supported local research and development of these products.^46^ The ban on GMO importation and cultivation lasted for 10 years. The Kenyan government lifted it in 2022, but the decision was quickly challenged in court, with petitions lasting two years before they were dismissed in November 2024.^47,48^

As of 2024, the National Biosafety Authority (NBA) in Kenya has approved over 50 GMO projects, the majority of which were for contained use in laboratories and greenhouses, approximately 15 for confined field trials (CFT), and three for commercial cultivation or environmental release.^48^ The latter is for Bt cotton, Bt maize, and cassava event 4046.^45,48,49^ Bt cotton and Bt maize were approved for commercialization in 2020 and 2022, respectively, while cassava event 4046 showed high resistance to cassava brown streak disease (CBSD) and increased marketable storage root yields compared to conventional cassava, progressing through confined field trials, national performance trials, and regulatory approval.^48,50,51^

Conclusively, there are reports that CFTs for GM gypsophila are complete, whereas those for sorghum, banana, and sweet potatoes are ongoing as of 2020.^46^

### Ethiopia

Ethiopia initially adopted a highly restrictive approach to genetically modified (GM) crops following the enactment of Biosafety Proclamation No. 655/2009. The legislation imposed stringent controls on the importation, development, use, and transboundary movement of GM materials, including requirements for prior authorization before transporting such materials through the country (Federal Democratic Republic of Ethiopia, Proclamation No. 655/2009). A major concern underlying this precautionary position was that adopting GM crops could undermine Ethiopia’s reputation in international markets as a supplier of organic agricultural products.^52^ This reputation has been particularly important for the marketing of coffee, spices, oilseeds, and other horticultural commodities.

A major policy shift occurred in 2015 with the enactment of the amended Biosafety Proclamation No. 896/2015 (Federal Democratic Republic of Ethiopia, Proclamation No. 896/2015), which relaxed some of the earlier restrictions and created opportunities for domestic biotechnology research. The revised policy initially permitted Ethiopian researchers to participate in the development and evaluation of GM non-food crops.^53^ This regulatory transition also helped create a more enabling environment for agricultural biotechnology research in Ethiopia, which hosts approximately 11 Consultative Group on International Agricultural Research (CGIAR) centers and associated programs.^54^

In 2018, the National Variety Release Committee (NVRC) approved the environmental release and commercialization of Bt cotton following two years of confined field trials.^54–56^ In the same year, the NVRC authorized confined field trials of genetically modified enset (*Ensete ventricosum*), an important food-security crop in Ethiopia.^54,55^

Ethiopia reached another major milestone in February 2025, when the NVRC approved the commercial release of three genetically engineered TELA maize varieties following extensive evaluation. These varieties were developed to provide resistance to stem borers and fall armyworm while also improving drought tolerance.^55^ The released varieties have reportedly demonstrated grain-yield advantages of up to 60% over conventional maize varieties under appropriate production conditions.

Despite this progress, the adoption of agricultural biotechnology in Ethiopia remains contested. Some civil-society organizations and activists have called for a return to the more restrictive provisions of the 2009 proclamation, arguing that expanding GM technology could increase foreign influence over the national seed sector. Consequently, public misinformation, limited awareness, competing stakeholder interests, and concerns about seed sovereignty continue to constrain biotechnology research and commercialization in the country.^54^

### Nigeria

A report by ISAAA^27^ ranked Nigeria as the sixth country in Africa to adopt GM crop cultivation and the first in the world to commercially release Bt cowpea.^16^ Nigeria established the National Biosafety Management Agency Act in 2015, and since then, it has approved over 30 genetically modified crop events.^57^

The National Biosafety Management Agency has approved four GM crops for commercialization in Nigeria: Tela maize, Bt cotton, soybeans, and Bt cowpea.^58^ In 2018, Bt cotton was approved for commercialization, farmer fields were established, and full commercialization was realized by 2020.^27^ The second GM crop to be released for open-field cultivation was the pod borer-resistant Bt cowpea SAMPEA 20-T variety.^16^ This released variety was developed through international collaboration under the supervision of the African Agricultural Technology Foundation (AATF). It involved the Network for Genetic Improvement of Cowpea for Africa (NGICA) and the Commonwealth Scientific and Industrial Research Organization (CSIRO) in Australia, the U.S. Agency for International Development (USAID), the African Agricultural Technology Foundation (AATF), the Danforth Plant Science Center, and the Rockefeller Foundation, scientists from the Institute for Agricultural Research (IAR) of Ahmadu Bello University, Zaria, Institutions in Ghana, Burkina Faso and Malawi.^31^ The adoption of Sampea 20-T significantly reduced farmers’ pesticide use and increased crop yield by about 20%, reducing dependence on imports.^59,60^

In 2021, Tela maize was approved for environmental release and field trials. GM maize was genetically engineered to improve resistance to fall armyworms and drought tolerance.^58^ Four Tela maize varieties were approved for commercialization, including SAMMAZ 72T, SAMMAZ 73, SAMMAZ 74T, and SAMMAZ 75T. The maize varieties have a very high yield prospect of up to 10 tons per hectare when grown under good agronomic practices.^58^

Some individuals and groups posit a hidden agenda to GM cultivation in the country and the takeover of the national seed sector by the west especially the US.^54,61^ These anti-GMO groups have advocated for legislative measures to restrict the cultivation or importation of GM crops.^61^ On the other hand, increasing sensitization campaigns from scientists, research organizations, farmers’ associations, as well as NABDA, through the National Orientation Agency (NOA) have increased Nigerians’ acceptance of genetically modified foods.^61^

### Uganda

As early as 2007, Uganda had already started a journey toward the commercial approval of GM crops.^62^ The first CFTs involving GM crops in Uganda were bananas resistant to black sigatoka disease.^62^ Thereafter, the Confined Field Trials (CFTs) for cassava varieties resistant to CBSD and drought-resistant maize were approved by the Uganda Biosafety Committee.

With the establishment of the National Agricultural Biotechnology Center (NABC) in 2013, significant progress has been made in Uganda. By 2023, more than 17 field experiments for genetically engineered crops have been approved. Confined Field Trials (CFTs) have been conducted for GM bananas enriched with vitamin A, and Cassava resistant to CBSD.^62,63^ Many studies on genetically modified maize have been conducted in Uganda with CFTs. Research has been conducted on herbicide- and late-blight-resistant potatoes.^63^

Even with the progress of CFTs for various crops in Uganda, the environmental release and ultimately adoption of the GM crops by the farmers cannot be achieved because the country lacks biosafety laws guiding the commercialization of GMOs.^62,64^ Additionally, health-related concerns, environmental impact and foreign control over food production have hindered the adoption of GM crops in the country.^65^

### Ghana

Ghana has progressed from establishing a comprehensive biosafety framework to researching genetically modified (GM) crops, implementing confined field trials, and approving selected GM products for cultivation and importation.^66–68^ This progress reflects Ghana’s commitment to harnessing modern biotechnology to enhance agricultural productivity, strengthen food security, and improve climate resilience while maintaining science-based oversight to safeguard human health and the environment.^66,69^

A major milestone in this development was the enactment of the Biosafety Act, 2011 (Act 831), which established the legal framework for the development, importation, environmental release, commercialization, and monitoring of genetically modified organisms (GMOs) and created the National Biosafety Authority (NBA) as the country’s regulatory body.^70^ The Biosafety (Management of Biotechnology) Regulations, 2019 (L.I. 2383) further strengthened the regulatory framework by providing detailed procedures for risk assessment, confined field trials, environmental release, importation, and post-release monitoring.^71^ Collectively, these legislative instruments have positioned Ghana among the few West African countries with a functional, science-based biosafety regulatory system.^66,67,72,73^

With this regulatory framework in place, Ghana initiated confined field trials of several GM crops, including Bt cowpea, Bt cotton, and nitrogen-use-efficient, water-use-efficient and salt-tolerant (NEWEST) rice, through the Council for Scientific and Industrial Research (CSIR) and its partners.^67,72,74,75^ These trials led to approval for environmental release and commercial cultivation, making it Ghana’s first genetically modified crop approved for cultivation.^76,77^ Commercialized as Songotra-T, the variety has shown promising early adoption, particularly in Northern Ghana, with farmers reporting higher yields, fewer insecticide applications, earlier maturity, and lower production costs than conventional varieties.^78,79^ However, widespread adoption remains constrained by limited certified seed, extension support, and farmer awareness, shifting the focus from regulatory approval to strengthening seed systems, extension services, and technology stewardship.^79,80^

In addition to domestic commercialization, Ghana has established a regulated pathway for the importation of GM commodities. In 2024, the National Biosafety Authority approved 14 genetically modified crops (eight maize and six soybean) for direct use as food, feed, and industrial raw materials under strict regulatory oversight.^68^ These approvals authorize importation and utilization but do not permit commercial cultivation within the country.^68,77^

Ghana has made significant progress in establishing a robust biosafety system and advancing the responsible use of agricultural biotechnology. Nevertheless, Bt cowpea remains the country’s only GM crop approved for cultivation, highlighting the need for continued investment in biotechnology research, regulatory capacity, public engagement, and seed system development to support the sustainable adoption of GM technologies.^66,73,77^

## National Biosafety Regulations for GM Release

Regulations are critical for attaining broad socio-economic goals such as safety, equitable income distribution, maintaining public trust, improving resource allocation efficiency, and protecting ownership rights. Biosafety rules are laws or recommendations that aim to restrict conduct. According to Komen et al.,^67^ biosafety legislation should allow countries to preserve the environment and public health while reaping the benefits of modern biotechnology.

Consequently, the Convention on Biological Diversity (CBD) established the legally binding Cartagena Protocol on Biosafety to help parties develop systems for the environmentally responsible management of contemporary biotechnology practices. The document centers on the transboundary movement of living-modified organisms (LMOs) and their potential impact on biodiversity. Angola, Chad, and Somalia lack regulatory frameworks, while Burkina Faso, Egypt, Ghana, Kenya, Mali, Namibia, Nigeria, South Africa, Uganda, and Zimbabwe have legal frameworks that enable them to regulate modern biotechnology to varying extents.^62^

The regulatory landscape for GM crops in several African countries demonstrates differing perspectives and attitudes about agricultural biotechnology.^62^ While some countries have used genetically modified (GM) crops to increase agricultural production and food security, others have implemented careful regulatory frameworks that prioritize rigorous safety assessments and risk management.^81^ For instance, South Africa has established a comprehensive legal framework that allows for the commercial production of select GM crops, indicating a very promising prognosis.^82^ On the other hand, some African nations still impose stringent laws or complete prohibitions because of concerns about possible negative impacts on the environment, public health, and socioeconomic conditions.^83^

Although nations like Egypt and Burkina Faso were early adopters of genetically modified (GM) crops in Africa, they have recently retracted their progress in this domain.^62^ In contrast, countries such as Ethiopia, Kenya, Ghana and Nigeria are just starting to integrate this technology into their agricultural practices.^84^ Nations like Tanzania, Uganda, and Zimbabwe have enacted more stringent policies, prohibiting the cultivation of certain GM crops.^62^ Nonetheless, the absence of harmonization in regulatory frameworks for biotechnological products across Africa hinders research initiatives, trade, and regional collaboration.

## Stakeholders’ Support for Acceptance and Commercialization of GM Crops

Government and Legislation: The success of a product in terms of commercialization and widespread adoption is often contingent upon the deliberate backing of the government. For GMOs, this support is reflected in the creation of well-defined and transparent regulatory systems for their approval and commercialization.^85^ Political commitment is further demonstrated through regulatory frameworks that promote public awareness via institutions, organizations, media outlets, and other channels to educate people about the advantages of GM crops.^86,87^ Bailey et al.^85^ highlighted the consequences of lacking domestic institutional capacity, which can lead to biosafety regimes that are absent, ineffective, or overly restrictive.

It is important to note that divergent biosafety policies among neighboring countries may complicate the trade and exchange of agricultural products, research partnership and, in some cases, generate regulatory or diplomatic tensions. For example, before Ethiopia enacted the amended Biosafety Proclamation No. 896/2015, genetically modified plants were reportedly found growing in border areas, possibly as a result of the cross-border movement of seed from Kenya, where regulations governing GM crops were comparatively more permissive at the time.^53^ A similar policy contrast exists in East Africa, where Kenya has adopted a more favorable regulatory position toward GM crops, while neighboring Uganda and Tanzania have maintained more restrictive approaches to their commercialization, including importation.^62,88^

These differences highlight the importance of regional coordination in biotechnology governance. Neighboring and trading-partner countries should work collaboratively to harmonize, or at least make compatible, their biosafety procedures for seed movement, product labeling, import approval, traceability, and border monitoring. Such cooperation would help prevent unintended cross-border movement of GM materials, reduce trade disruptions, and ensure that differences in national biotechnology policies do not undermine longstanding agricultural and economic relationships.

Civil Societies, non-governmental and international organizations: Numerous organizations typically resist commercialization, thereby categorizing them as the Anti-GMO group. In South Africa, for example, this group comprises several small organizations that collaborate to form a cohesive opposition. One strategy to amplify their presence is to use public petitions, which they then present to various forums. Notable organizations within this group include the South African Freeze Alliance on Genetic Engineering (SAFeAGE), the African Center for Biosafety (ACB), Earthlife Africa (ELA), Biowatch South Africa, and Greenpeace South Africa.^89^ The anti-GMO lobbyists raise several concerns, including the safety of genetically modified (GM) crops for consumers, the impact of GM crops on the environment and biodiversity, the lack of consumer choice due to inadequate product labeling, and potential adverse socio-economic effects.^89^ Smyth et al.^87^ linked the rejections of GM maize imports by some southern and East African countries to influence from European and North American environmental organizations.

Farmers: The application of genetically modified (GM) technology has drawn significant criticism for its predominantly top-down approach, which tends to benefit biotechnology companies rather than adopting a bottom-up strategy that prioritizes farmers as primary beneficiaries. Moreover, this technology often marginalizes small-scale farmers while favoring large-scale agricultural enterprises.^90^ Elements of truth reside in this claim as private companies didn’t forecast much revenue for African farmers, the majority of whom are poor resource and smallholder farmers who save seeds over generations, have weak intellectual property rights protection, and maintain limited structured seed markets.^90,91^ This disparity is evident in the types of GM crops available and the associated seed costs for farmers. Even if small-scale farmers express interest in adopting GM technology, the financial burden of GM inputs remains prohibitive for those with limited resources.^90^ Although there is a moral rationale for the high costs, given the comprehensive expenses involved in the production of these crops by biotechnology firms, some experts argue that the adoption of GM crops by large-scale farmers could lead to increased production, thereby reducing prices and enhancing food availability for economically disadvantaged populations.^92^ While this perspective, rooted in a Western viewpoint, suggests that food security can be achieved through the inexpensive surplus production of large-scale farmers, it is primarily applicable in contexts such as South Africa, where large-scale farmers contribute approximately 75% of food production. Conversely, in West African nations like Nigeria, where subsistence farmers are responsible for about 70% of food consumption, focusing solely on large-scale farmers may be inappropriate. Given the nuanced impacts of GMOs on both types of farmers,^92^ it is crucial to consider the needs of both groups. Dowd-Uribe et al. 2023 proposed a public-private-partnership as a panacea for the adoption of GM crops by most smallholder African farmers. This initiative would not only address concerns, dispel misconceptions, and provide accurate information to the public, but also create a shift in traits tailored toward poor resource farmers.^61^

Biotech Industries: Biotech companies are often viewed as being primarily profit-oriented, with less regard for human health and environmental impacts.^75,90^ The advantages of GMOs, as promoted by these companies, are perceived as overstated, with the intent to dominate the market and take advantage of small-scale farmers.^93,94^ This perception of biotech firms further solidifies public skepticism and strengthens resistance to the adoption and commercialization of their products. So, how can this perception be altered? Building trust is essential. Even if the public remains skeptical of the companies’ evidence in the long term, these companies must strive for transparency in the detailed process of testing and commercializing their products. While promoting the benefits of GM crops, they should also provide credible reports from independent research that assess potential risks, allowing the public to evaluate them. This transparency would enable the public to make informed decisions about whether to accept these products or not.

Scientists and Academia: The scientific community and academia represent two pivotal groups of intellectuals in the discourse surrounding genetically modified (GM) crops. As intellectuals, they are presumed to possess comprehensive knowledge of this subject. In many instances, scientists’ perspectives are influenced by the organizations with which they are affiliated and are perceived as proponents of GMOs, largely due to their associations with companies or institutions involved in the production of transgenic crops often with incentives to conduct biotech research.^95^ Consequently, the public often assumes that scientists’ views are biased, leading to reduced acceptance. Conversely, academia is regarded as the most credible stakeholder in the public discourse on agricultural biotechnology in developing countries^92^. However, the situation differs in Europe, where academia is perceived to align with biotech industries for commercial reasons. Given the high regard in which both scientists and academia are held due to their expertise on GM crops, it is anticipated that they will provide an impartial analysis of the risks and benefits associated with GM commercialization and offer balanced information to the public.

Consumers: One reason consumers reject GM products is that farmers are the target beneficiaries. Most GM crops bear traits that serve exclusively for agronomic purposes, hence benefiting only farmers. For instance, Bt crops like maize, soybean, cotton, etc., are targeted to confer resistance to insect attack and invariably boost yield in farmers’ fields. No increase in nutrient constituents or decrease in toxic compounds are associated with these transgenic crops. Again, GM technology is a bottom-up rather than a top-down approach. It can be safely assumed that when these products offer beneficial consumption properties to end consumers, conveyed with proper enlightenment, they will most likely be endorsed and accepted by the public. Furthermore, raising awareness of the potential benefits of GM crops will proportionally increase positive perception and acceptance.^96^ Studies from South Africa,^97^ Kenya,^98^ Tanzania,^99^ Nigeria, and Ghana,^96^ highlighted the importance of public awareness in shaping positive perceptions and acceptance of GM crops. Oladipo et al.^100^ reported the benefits of leveraging credible media outlets to sensitize the public of genetic modification techniques and safety of the final product. Furthermore, “premised on right to know” proper labeling helps consumers decide whether to buy a product or not for its GM content.^66,101^

## Prospects of Transgenic Crops to the Continent

Plant breeders are increasingly developing improved crops to address the diverse production and end-user needs of African agriculture. Transgenic technology offers opportunities to enhance crop productivity, quality, resilience, and nutritional value by introducing specific traits that may be difficult or time-consuming to achieve through conventional breeding.^102–106^ Beyond established traits such as insect and herbicide resistance, virus resistance, and modified fruit ripening, future applications are expected to focus on resistance to emerging pests and diseases, environmental stresses, and nutritional deficiencies.^106–108^ Gene stacking and advances in omics technologies could further facilitate the development of crops with multiple desirable traits.^109–111^

Climate resilience is particularly important for African agriculture, where drought, erratic rainfall, salinity, and increasing pest and disease pressures pose significant threats to crop production.^112–114^ Genetic engineering provides opportunities to develop crops with improved tolerance to these stresses and enhanced nutrient-use efficiency.^67,115–117^ For example, Water Efficient Maize for Africa (WEMA) and its subsequent TELA maize products demonstrate the potential of combining drought tolerance with insect resistance to improve yield stability under challenging conditions.^118–120^ Such technologies could be particularly valuable to smallholder farmers who depend heavily on rain-fed agriculture.^121^

Advances in genome editing are further expanding opportunities for crop improvement. Technologies such as CRISPR/Cas and TALENs enable targeted modification of genes and biological pathways associated with disease resistance, stress tolerance, and nutritional quality.^122–124^ When integrated with conventional breeding and genomic approaches, these technologies can accelerate the development of improved varieties of important African staple crops such as maize, cassava, sorghum, and millet.^123,125,126^ However, genome-edited crops should be distinguished from transgenic crops because some genome-editing approaches do not involve the introduction of foreign DNA.^123,127,128^

Another important prospect is nutritional improvement through biofortification. Genetic engineering can enhance the concentration or bioavailability of nutrients such as provitamin A, iron, zinc, and essential amino acids in staple crops.^129–131^ Examples such as Golden Rice and nutrient-enhanced cassava and sweet potato illustrate the potential of biotechnology to complement conventional biofortification and help address micronutrient deficiencies.^132–135^

Realizing these opportunities will require continued collaboration among African research institutions, universities, international organizations, and biotechnology partners to strengthen research capacity, facilitate technology transfer, and support the responsible development and deployment of improved crops.^126,136^ Realizing these opportunities will require sustained collaboration among African research institutions, universities, international organizations, and biotechnology partners to strengthen research capacity, facilitate technology transfer, and support the responsible development and deployment of transgenic crops.^126,136^ Equally important are robust biosafety and regulatory systems, effective seed delivery networks, and meaningful stakeholder engagement to promote safe and sustainable adoption of these technologies in Africa.^16,66,137^ Ultimately, transgenic technology should be integrated with conventional breeding, genomics, genome editing, and sustainable crop management to develop productive, nutritious, and climate-resilient crops adapted to African farming systems.^65,126^

## Success Stories of GMOs and Lessons Learned from the Developed World

The success stories of genetically modified organisms (GMOs) in the developed world offer valuable insights into their potential benefits and the lessons learned from their implementation. Over the past two decades, GMOs have demonstrated significant socioeconomic and environmental advantages, particularly in North America and Europe. These benefits include increased agricultural productivity, reduced pesticide use, and enhanced food security, which are critical for addressing global challenges, such as climate change and population growth.^138–140^

In the United States, adopting GM crops has brought substantial economic benefits to farmers. For instance, the introduction of Roundup Ready soybeans has been associated with higher yields and lower production costs, thereby enhancing farm-level welfare.^141^ Similarly, studies show that the diffusion of GMOs has reduced pesticide use, which not only lowers costs for farmers but also supports environmental sustainability through conservation tillage practices.^139,140^ These outcomes illustrate GMOs’ potential to support sustainable agricultural practices while meeting the food demands of a growing population.

Moreover, regulatory frameworks in the developed world have played a crucial role in integrating GMOs into agriculture. The United States uses a relatively streamlined regulatory process that allows the commercial release of GM products based on rigorous safety assessments focused on allergenicity, digestibility, and toxicity.^142^ In contrast, the European Union has adopted a more precautionary approach, leading to stricter regulations and labeling requirements for GM foods.^19^ This divergence in regulatory strategies highlights the importance of context-specific approaches to GMO governance, as the EU’s stringent regulations may hinder innovation and adoption of beneficial technologies in agriculture.

The lessons learned from the developed world regarding public perception and education regarding GMOs are also noteworthy. Research indicates that increasing public knowledge of GM technology can positively influence attitudes toward genetically modified foods.^143^ Educational interventions that enhance the understanding of the science behind GMOs can help mitigate skepticism and foster acceptance among consumers.^144^ This underscores the necessity of transparent communication and public engagement in the discourse surrounding GMOs, which is essential for their broader acceptance and utilization.

Conclusively, the success stories of GMOs in the developed world underscore their potential to enhance agricultural productivity, promote environmental sustainability, and improve food security. The experiences gained from these regions provide valuable lessons for other countries regarding the adoption of GMO technology, particularly regarding regulatory frameworks, public education, and stakeholder engagement. As the global agricultural landscape continues to evolve, insights derived from the developed world’s experiences with GMOs will be instrumental in shaping future policies and practices.

## Conclusion

Genetically modified (GM) crops offer transformative potential for addressing Africa’s pressing agricultural challenges, including low productivity, pest and disease pressures, and climate-induced stress. Success stories from countries such as South Africa, Nigeria, Kenya, and Sudan demonstrate the tangible benefits from the adoption of GM crops. In South Africa, commercial cultivation of Bt maize and cotton has led to increased yields, reduced input costs, and improved farmer incomes over two decades. Similarly, Sudan has scaled up Bt cotton cultivation from 20,000 hectares in 2012 to 192,000 hectares in 2017, contributing significantly to national agricultural output. In 2022, the Ghana National Biosafety Authority approved the environmental release of GM cowpea resistant to the Maruca pod borer, while in 2024, it approved the commercialization of fourteen (14) GMO products comprising eight (8) and six (6) events for maize and soybeans, respectively.

Nigeria made history as the first country globally to approve and release Bt cowpea (Sampea 20-T), a breakthrough that drastically reduced pesticide uses and increased yields by approximately 20%. The country has also commercialized Tela maize, which combines drought tolerance with resistance to fall armyworm, traits critical for climate resilience. After lifting a decade-long GMO ban, Kenya has advanced the commercialization of Bt cotton and Bt maize, while also conducting trials for virus-resistant cassava and biofortified crops like vitamin A-enriched bananas.

Despite these achievements, adoption remains limited and uneven across the continent. Burkina Faso, once a leader in GM cotton, withdrew its Bt varieties due to fiber quality concerns, highlighting the importance of locally adapted breeding and comprehensive trait integration. Countries like Uganda and Tanzania have invested in field trials but face delays due to absent or restrictive biosafety laws. Egypt, an early adopter, suspended GM maize commercialization amid public backlash fueled by misinformation.

Public skepticism, regulatory fragmentation, and fears of market exclusion continue to obstruct wider adoption.^145–149^ This hesitancy persists despite affirmations by global health authorities like WHO and FAO, which maintain that GM crops approved through rigorous safety evaluations pose no greater risk than conventional crops.^150,151^

It is worth mentioning that there is ongoing GM research on stem borer-resistant, streak virus-resistant and drought-tolerant maize; weevil-resistant sweet potato; nematode- and weevil-resistant and biofortified banana; virus-resistant and biofortified cassava; biofortified sorghum, pod borer-resistant cowpea; nitrogen-use-efficient, water-use-efficient and tolerant rice, across different countries in Africa.^152^

To harness the full potential of GM technology, Africa must adopt science-based, harmonized biosafety frameworks and foster transparent communication about the risks and benefits of GMOs. Stakeholder collaboration, including farmers, researchers, governments, and biotech industries, is essential for building public trust and ensuring equitable access to biotechnology. Strengthening national research capacity and promoting regional cooperation will also ensure that GM solutions are tailored to local agro-ecological and socio-economic realities. When strategically integrated into broader food system reforms, GM crops can play a pivotal role in achieving sustainable agriculture, enhancing food security, and reducing poverty across the African continent.

## Data Availability

No primary datasets were generated for this review article. The work is based on analysis and synthesis of previously published studies, all of which are cited in the reference list.
